# Investigating Racial Disparities in Cancer Crowdfunding: A Comprehensive Study of Medical GoFundMe Campaigns

**DOI:** 10.2196/51089

**Published:** 2023-12-12

**Authors:** Xupin Zhang, Jingjing Wang, Jamil M Lane, Xin Xu, Silvia Sörensen

**Affiliations:** 1 School of Economics and Management East China Normal University Shanghai China; 2 Department of Environmental Medicine and Public Health Icahn School of Medicine at Mount Sinai New York, NY United States; 3 Warner School for Education and Human Development University of Rochester Rochester, NY United States

**Keywords:** crowdfunding, racial discrimination, GoFundMe

## Abstract

**Background:**

In recent years, there has been growing concern about prejudice in crowdfunding; however, empirical research remains limited, particularly in the context of medical crowdfunding. This study addresses the pressing issue of racial disparities in medical crowdfunding, with a specific focus on cancer crowdfunding on the GoFundMe platform.

**Objective:**

This study aims to investigate racial disparities in cancer crowdfunding using average donation amount, number of donations, and success of the fundraising campaign as outcomes.

**Methods:**

Drawing from a substantial data set of 104,809 campaigns in the United States, we used DeepFace facial recognition technology to determine racial identities and used regression models to examine racial factors in crowdfunding performance. We also examined the moderating effect of the proportion of White residents on crowdfunding bias and used 2-tailed *t* tests to measure the influence of racial anonymity on crowdfunding success. Owing to the large sample size, we set the cutoff for significance at *P*<.001.

**Results:**

In the regression and supplementary analyses, the racial identity of the fundraiser significantly predicted average donations (*P*<.001), indicating that implicit bias may play a role in donor behavior. Gender (*P*=.04) and campaign description length (*P*=.62) did not significantly predict the average donation amounts. The race of the fundraiser was not significantly associated with the number of donations (*P*=.42). The success rate of cancer crowdfunding campaigns, although generally low (11.77%), showed a significant association with the race of the fundraiser (*P*<.001). After controlling for the covariates of the fundraiser gender, fundraiser age, local White proportion, length of campaign description, and fundraising goal, the average donation amount to White individuals was 17.68% higher than for Black individuals. Moreover, campaigns that did not disclose racial information demonstrated a marginally higher average donation amount (3.92%) than those identified as persons of color. Furthermore, the racial composition of the fundraiser’s county of residence was found to exert influence (*P*<.001); counties with a higher proportion of White residents exhibited reduced racial disparities in crowdfunding outcomes.

**Conclusions:**

This study contributes to a deeper understanding of racial disparities in cancer crowdfunding. It highlights the impact of racial identity, geographic context, and the potential for implicit bias in donor behavior. As web-based platforms evolve, addressing racial inequality and promoting fairness in health care financing remain critical goals. Insights from this research suggest strategies such as maintaining racial anonymity and ensuring that campaigns provide strong evidence of *deservingness.* Moreover, broader societal changes are necessary to eliminate the financial distress that drives individuals to seek crowdfunding support.

## Introduction

### Background

In recent years, medical crowdfunding has become a popular way for people to raise funds for medical expenses, treatments, surgeries, and other health care needs. Platforms such as GoFundMe and YouCaring have made it easy for individuals to set up and share campaigns. Crowdfunding for medical expenses has become a popular method of covering health care costs in response to unmet medical needs owing to a lack of adequate insurance coverage [1]. Indeed, approximately 22% of adults in the United States reported that they have donated to medical campaigns on GoFundMe. As of 2021, an estimated US $650 million, which constitutes approximately one-third of the total funds raised on the GoFundMe platform, was directed toward medical campaigns [2].

Currently, most medical crowdfunding campaigns convey the patient’s situation and funding needs through textual descriptions, which can vary from a few words to tens of thousands in length. Cover images are mainly of 2 types: those depicting daily life settings and those set in medical environments. These images not only evoke empathy from potential donors but also enhance the campaign’s authenticity and credibility. The inherent information asymmetry on crowdfunding platforms poses a significant challenge in accurately evaluating the genuine intentions of fundraisers, thereby encompassing the potential for fraudulent campaigns designed to deceive donors for financial gain [3]. For most potential donors, direct communication with the fundraiser is limited, and tracking treatment progress is difficult. Faces have the remarkable ability to swiftly convey information such as an individual’s gender, state of health, ethnic heritage, and recovery progress, thereby influencing how potential donors perceive and interact with the fundraiser [4]. In this context, a fundraiser’s race is easily identifiable and can readily activate donors’ stereotypes and influence their behavior. Moreover, the growth of medical crowdfunding campaigns activates and perpetuates prevailing societal beliefs about who does and does not deserve charity. In the United States, these practices are deeply ingrained in the history of racial and gender oppression, where certain populations are routinely deemed unworthy of social support and charitable aid [5]. As Noble and Tynes [6] pointed out, the prevailing internet culture is both shaped by and consequently most benefits those who are White, male, and capitalist.

Examining racial differences in medical crowdfunding is essential for understanding disparities in access to crowdfunding assistance between White individuals and other races. Although these differences may be interpreted as evidence of discrimination, in this study we also explored alternative and additional explanations. Research in broader medical crowdfunding suggests such disparities, but crowdfunding in cancer treatment may differ from other types of crowdfunding, given variations in patient blaming and stigma among different cancers and between cancers and other illnesses and accidents [7,8]. In addition, this study helps to identify how social mechanisms can contribute to racial gaps in cancer crowdfunding and promote greater social equity. If evidence indicates that persons of color might experience systematic discrimination in cancer crowdfunding and have a harder time attracting donors, this supports previous contentions that medical crowdfunding could potentially exacerbate existing racial disparities and health inequalities, both of which are crucial issues that require attention [9].

This study used DeepFace [10] for racial recognition. We examined medical crowdfunding campaigns on the GoFundMe platform to investigate the impact of fundraisers’ race on their fundraising performance and to make recommendations to address the problem of insufficient medical resource distribution among communities of color. This study expands crowdfunding and racial discrimination research by demonstrating race’s predictive value for multiple fundraising outcomes. In addition to the direct effects of race on crowdfunding success, we examined racial differences in combination with other types, such as gender, age, and the racial composition of the neighborhoods of campaign initiators. Finally, our study introduces new performance indicators, such as average donation amount and donation counts, enabling a deeper exploration of the discrimination characteristics.

In summary, this study is primarily motivated by the growing concern over racial disparities in medical crowdfunding, specifically within the context of cancer crowdfunding on the GoFundMe platform. This study aims to investigate and shed light on the racial inequalities that exist in fundraising outcomes within this domain. Several hypotheses have been proposed to address this aim. First, it is hypothesized that campaigns led by individuals from different racial backgrounds will exhibit significant disparities in fundraising outcomes. Second, the study posits that the disclosure of racial information within campaign descriptions will have a notable impact on the success of fundraising efforts. Finally, the research hypothesis suggests that the racial composition of the neighborhood of campaign initiators will exert an influence on fundraising outcomes. By exploring these hypotheses, this study aims to contribute to a deeper understanding of the presence and underlying mechanisms of racial disparities in the realm of cancer crowdfunding, thus furthering discussions on equity and fairness in health care financing.

### Literature Review

Medical crowdfunding is a rapidly growing and largely unregulated industry that transforms how Americans receive social and financial assistance for chronic and acute health problems. Many people turn to medical crowdfunding when other health care coverage and social safety nets fail [11]. Researchers have questioned whether medical crowdfunding can effectively, efficiently, or equitably allocate health care resources to those who need them most [12]. One reason for this concern is the potential for discrimination within medical crowdfunding and the perpetuation of racial inequities in health and health care because of disparities in crowdfunding.

According to the social identity theory, individuals tend to categorize themselves into certain groups and distinguish themselves from other groups. This sense of belonging can lead to individuals providing more support and assistance to members of their group. In addition to the social identity theory, the homophily framework provides another mechanism to explain why people of the same race would show preferential bias. Homophily is the tendency of individuals to associate with others based on shared characteristics [13,14]. Activist choice homophily suggests that the connection between 2 individuals is not only based on their similarities but also their shared experiences of social barriers owing to their common group identity [15]. Therefore, it can be inferred that in crowdfunding, donors may be more inclined to support fundraisers with the same racial background, which establishes a theoretical basis for examining racial discrimination in the context of medical crowdfunding.

Racial discrimination remains a persistent issue in both offline markets and web-based platforms across the United States. For example, in offline markets, Black applicants are less likely to be approved for mortgage loans than their White counterparts despite having comparable credit and financial characteristics [16,17]. If approved, Black applicants are subject to higher interest rates, which is a byproduct of systemic racism. In the auto-leasing market, similar insidious discrimination has occurred, with Black and Hispanic applicants’ loan approval rates being 1.5 percentage points lower even after controlling for creditworthiness [18]. Furthermore, racial discrimination has also infiltrated web-based marketplaces; for example, discrimination against Asians and Asian-Americans on Facebook [19], including hate crimes, microaggressions, and vicarious discrimination [20], as well as on Twitter [21], Airbnb [22], and web-based ridesharing services [23].

The fact that significant biases persist in these areas establishes a motivation for examining racial discrimination in the context of cancer crowdfunding. There are 2 types of crowdfunding campaigns in which discrimination may occur: donation crowdfunding and reward crowdfunding. Donation crowdfunding is a charitable act that does not require returns, as represented by medical crowdfunding. Reward crowdfunding takes the form of in-kind, shares, and debentures as the return, which are issued to the backers after the success of the campaign. Examples of such platforms are Kickstarter and Indiegogo [24].

Most current research evaluating racial discrimination has focused on reward crowdfunding, mainly on Kickstarter, a US website that raises funds for return crowdfunding campaigns. For instance, Younkin and Kuppuswamy [25] discovered that African American men have a notably lower likelihood of receiving funding compared with their White counterparts with similar backgrounds, and potential backers tend to evaluate identical campaigns as being of lower quality when they perceive the founder to be an African American man. In addition, studies have further demonstrated that racial cues of fundraisers may affect funding outcomes, and racial anonymity can lead to higher success rates to some extent [24]. In addition, by examining the demographic attributes of donors from their names, residential locations, and other publicly accessible data, Clark et al [26] determined that donors with the same demographic characteristics as the fundraiser contribute more frequently than expected, forming a crowdfunding community. In summary, previous studies demonstrate that the racial background of fundraisers plays a crucial role in determining funding outcomes in reward crowdfunding.

Several studies have suggested that racial discrimination may exist in medical crowdfunding as well, illustrating that fundraiser racial background and social factors such as social class, educational background, and social network reach may influence fundraising goal completion and funds raised. In an exploratory cross-sectional study categorizing 637 medical crowdfunding campaigns from GoFundMe and donors by race, gender, age, and relationship about crowdfunding characteristics and outcomes, White groups exhibited a substantial advantage in medical crowdfunding [27], and African American fundraisers received significantly lower mean donations. Similarly, Barcelos [28] examined 410 medical crowdfunding initiatives with transgender needs as a fundraising goal. Their findings revealed that most web-based initiatives were dedicated to financing chest surgeries for young, White, binary gender–identifying transgender men in the United States. Evidence also suggests that bias against older persons and women is prevalent, with those groups being considerably disadvantaged in terms of funding outcomes, particularly in initiatives for personal education and health needs [29].

Successful medical crowdfunding requires a certain sophistication in understanding and describing medical terms and the effect of illness on the individual, which often results from education and health literacy. Davis et al [30] suggested that the 827 most successful medical crowdfunding campaigns in 2020 represent higher rates of White populations and reflect the types of illnesses and accidents that most frequently affect these populations. Using visually appealing, well-crafted storytelling, these campaigns reflect educational privilege and garner higher donation amounts.

Whereas some authors hypothesize that narratives of deservingness or simple interpersonal racial discrimination play a role in these disparities [24,30], others propose that racial and ethnic differences in donor network financial capacity play a considerable role in differences in campaign success [31,32]. During the COVID-19 pandemic, people from wealthier counties with higher levels of education, compared with people living in areas with lower income and education, were not only more likely to start new campaigns when they experienced health and economic crises but also received more funding [31]. On the basis of the names and geography of Facebook friend networks that reflect probable donor pools, Igra [32] reported that in a geographically stratified sample of 2618 campaigns, racial and ethnic differences in donor network financial capacity played a considerable role in differences in campaign success. Indeed, he argued that racial disparities in fundraising success may be the indirect result of systemic racism responsible for lower financial capital in the social networks of Black and Hispanic Americans rather than direct interpersonal prejudice. Our study sought to add information to better understand this question.

On the basis of the literature, we test the following hypotheses:

1. The race of the fundraiser has a significant impact on the number of fundraising contributors, and when the fundraiser is White, there is a higher number of donors.
2. The race of the fundraiser plays a significant role in determining the average amount raised, and when the fundraiser is White, the average donation per person is higher.

The race of fundraisers has a significant influence on fundraising outcomes, with White fundraising groups having a higher success rate.

Three attributes distinguish this study from previous works. First, we examine racial-ethnic disparities specifically about cancer crowdfunding, rather than all areas of health and financial crisis. Controlling the reason for fund requests has the advantage of limiting biases related to the stigma attached to pure financial crises and other causes. Second, our analyses include 3 funding outcomes: donation counts, average donation amount, and funding success compared with goal amounts. Third, rather than extrapolating race and ethnicity from names, and locations of fundraisers [1], we used artificial intelligence–driven facial attribute analysis to determine the most likely racial category of the campaign beneficiary. The visual images also generated donors’ first impressions. Using this approach, we can expand the racial categories to 4, thus providing more detail in our analysis than in previous studies.

## Methods

### Data

The sample for this study was from the largest American medical crowdfunding platform, GoFundMe. In addition to hosting a variety of fundraisers, including general medical and cancer-related ones, this platform aims to be a medium for organizations to promote racial justice and commemorate and raise awareness about racial injustice and is closely linked to prominent movements opposing nationalism and anti-Black activism [33,34]. The cancer crowdfunding campaigns included in the study were active between January 1, 2019, and July 11, 2021, all within the United States, focusing specifically on raising funds for cancer treatment. GoFundMe does not mandate that fundraisers specify the cancer type; therefore, a cancer lexicon was developed based on cancer classifications defined by The American Society of Clinical Oncology. Cancer types were then categorized using keyword matching in the campaign descriptions. The sample in this study matched a total of 90 cancer types, of which more than half (54,868) of the crowdfunding campaign descriptions did not involve specific cancer types. The top 5 cancers and their corresponding campaign numbers are presented in Table 1.

**Table 1:** Cancer type and item quantity (top 5).

Cancer type	Item quantity
Brain tumor	8736
Breast cancer	6087
Kidney cancer	3677
Lung cancer	3246
Bone cancer	3005

Because that the platform does not require the content of the uploaded images, this study removed the images that are not portrait or portrait images where racial identity information could not be determined. This approach enabled the acquisition of a sample that included visual racial information, which could later be compared with the sample without racial information used in the study. This resulted in a final sample size of 104,809 cancer campaigns with sufficient information to categorize the campaign initiators by race. This study focuses on the racial information contained in the cover images of the medical crowdfunding campaigns, not self-identification because images generally shape the donor’s first impression. The primary sample data were categorized and examined, encompassing fundamental campaign details (such as cover images, zip codes, campaign descriptions, and fundraising goals) as well as performance outcome data (including donation counts and average donation amount). In addition, as a comparison sample with launch time and country of origin comparable with the racially categorized sample, 50,000 racially anonymous campaigns (featuring nonhuman images on the cover) were collected.

### Image Recognition

Racial information in medical crowdfunding campaigns can be identified mainly by extracting campaign descriptions and portrait image information. Because images are generally the donor’s first impression, racial information in the images is extracted for further exploration. We used DeepFace, a Python-based framework for face recognition and facial attribute analysis. DeepFace uses state-of-the-art models such as VGG-Face, Google Face Net, and Open Face, with an accuracy rate of 97.53% [35,36]. It is important to note that the accuracy of facial attribute analysis is limited by the quality of its machine learning training data. The immense variability in human facial appearance is often not well represented in the training data, leading to flaws in the algorithms when recognizing individuals with darker skin tones [37-39]. Recent research has shown some improvements in reducing bias; however, accurately assessing bias remains a challenge [40-42]. Most of this research, however, has focused on correctly identifying individual faces rather than categorizing them by race, and although there has been some discussion of the inaccuracy of ethnicity identification, categorization by race appears to be less controversial [43]. Given the large volume of data involved in our study, comprising a total of 543,143 crowdfunding records, manual identification of the race of individuals in the images was not feasible, and any errors in identification were unlikely to significantly impact the subsequent analysis with the large sample size. Therefore, we selected DeepFace as a tool for image information extraction. In addition to racial information, gender and age may also affect donor behavior and are also extracted by image recognition. The gender includes man and woman, and the race includes (1) Asian, (2) American Indian or Alaska Native, (3) Black, and (4) White.

### Variables

The dependent variables are 3 crowdfunding campaign performance outcomes: donation counts, average donation amount, and funding success. The donation count is the number of unique donations received by the fundraiser. The average donation amount is calculated by dividing the total donation amount by the number of donations made to the campaign 
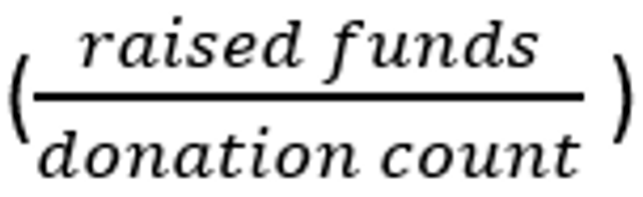
. The funding success refers to the amount of funds relative to the goal amount, with the variable recorded as 1 if the fundraising amount meets or exceeds the goal amount and 0 otherwise.

The primary independent variable in this study is the fundraiser’s race, indicated first as a binary variable with the White race labeled as 1 and other races labeled as 0. Of the 104,809 samples, 62,397 (59.53%) were identified as White. We controlled for 6 covariates based on existing crowdfunding research findings. For a more fine-grained analysis, we labeled racial categories as Asian, American Indian or Alaska Native, Black, and White.

Previous studies have demonstrated that women face disadvantages in crowdfunding [15,44]. The fundraiser’s gender, determined through image identification, was labeled men as 1 and women as 0; 73,663 (70.28%) of the samples were men. Although this percentage may seem high considering the general demographics of the population, it is important to note that men have a higher likelihood of being diagnosed with cancer than women [45]. Thus, it was representative of the overall population of incident cancer cases.

A fundraiser’s age can influence perceived credibility and potential donor support [46]. The age of the participants in this study’s sample ranged from 12 to 70 years, as revealed by the image recognition results.

Geographic factors play a significant role in shaping the outcomes of crowdfunding [47]. We obtained county-level data by machining the US Federal Information Processing Standards and ZIP code data sets with the fundraiser’s location data. Racial data were also acquired from the National Bureau of Economic Research using US intercity county population data by age, gender, race, and Hispanic origin, and the variable percentage of White people in the US county (number of White people/total population) was successfully extracted.

Textual information strongly correlates with crowdfunding outcomes [48]. The campaign description length, referring to the word count in the campaign’s textual description, provides an essential source of information for potential donors.

As the monetary target set by the fundraiser, the goal amount has been shown to impact crowdfunding success, with smaller goals more likely to receive support and achieve their targets [47]. Owing to the large range of this variable compared with other variables, logarithmic transformation was used for subsequent regression analyses.

In addition to the direct effects of race on crowdfunding success, there may be intersectionality effects with racial differences exacerbated by other types of social identities, such as gender and age [49]. Finally, the racial composition of the neighborhoods of campaign initiators may also affect their social network and potential respondents to their campaign. Thus, regression analyses of interaction terms of several other predictors (gender, age of fundraiser, and the proportion of White people in the county) with race were conducted (see the *Statistical* *Analysis* section). Tables 2 and 3 present the summary statistics for the main variables.

**Table 2:** Continuous variable summary statistics (N=104,809).

Variable	Values, mean (SD; range)
Age (years)	33.09 (6.82; 12-70)
Local White proportion	0.786 (0.16; 0.113-0.993)
Length of campaign description	1609 (1575; 0-32,078)
Goal amount	21,866 (985,620; 1-300,000,000)
Donation counts	52.52 (311.9; 273-378)
Average donation amount	82.81 (71.91; 0-5138)

**Table 3:** Discrete variable summary statistics (N=104,809).

Variable (label)		Value, n (%)
**Race**		
	White (1)	42,412 (40.5)
	People of racial and ethnic minorities (0)	62,397 (59.5)
**Gender**		
	Man (1)	31,222 (29.8)
	Woman (0)	73,587 (70.2)
**Funding success**		
	Success (1)	92,471 (88.2)
	Failure (0)	12,338 (11.8)

### Statistical Analysis

We examined 3 crowdfunding performance variables, including average donation amount, donation counts, and funding success. We used Stata (version 15.0; StataCorp) [50] to analyze the data.

### Data Preparation

When examining the effect of race on performance, potential differences in beneficiary characteristics may lead to significant bias [51]. For example, if White and individuals from other racial groups differ systematically in terms of their education level or income, these differences may affect their crowdfunding performance, leading to biased results [45]. coarsened exact matching (CEM) is a nonparametric matching technique that improves the comparability between 2 data sets by controlling the effects of confounding factors in the observed data on evaluation outcomes. Its primary objective is to maintain a balanced distribution of control variables in both the treatment and control groups [52-54]. Even though there was no treatment group in this study, the sample of White campaign initiators could be conceptualized as one, whereas the sample of campaign initiators from other racial backgrounds can be treated as a control group. Thus, the matching weights of the CEM were used to balance the effects of the covariates. For the independent variable fundraiser race, there were 62,397 treatment group observations representing the White campaign initiators and 42,412 control group observations representing campaign initiators from other racial backgrounds in the sample. There were 44,546 matches obtained for the White group sample and 33,007 matches for the sample from other racial groups. Specifically, in this study, we used Stata software to perform matching and the CEM method to balance the effects of the covariates. We first defined the covariates that needed to be balanced, and then we did the matching. Through CEM matching, 27,256 observations that would have affected the balance between the treatment and control groups were eliminated, making the results of the regression more meaningful.

### Steps in the Analysis

To better estimate the effect of fundraiser race on crowdfunding performance, we first investigated whether there were differences between those using racial identifiers in images and those who did not. We then addressed the possibility that medical crowdfunding programs differ systematically between White people and other ethnic groups.

The dependent variables for average donation amount and donation number were analyzed using linear regression, whereas the dependent variable for funding success was analyzed using logistic regression. The primary independent variable was the race of the fundraiser, with covariates, including the fundraiser’s gender and age, the local White population proportion, the length of the campaign description, and the fundraising goal amount.

In addition, regression analyses with interaction terms of several other predictors with race were conducted. These variables were (1) the gender of the fundraiser, (2) the age of the fundraiser, and (3) the proportion of White people in the county. The interaction terms were race×gender, race×age, and race× proportion of White people in the county.

### Ethical Considerations

In accordance with the East China Normal University's Institutional Review Board policy, our study did not require an ethics review. This exemption is based on the fact that our research solely utilized publicly available data. Consequently, no submission to the Research Ethics Board was necessary for this particular study.

## Results

### Regression Results

First, using CEM matching, 27,256 observations that could potentially impact the balance between the White and other racial groups were excluded. We took this step to ensure greater practical significance and to minimize unnecessary errors in the regression analysis. Table 4 presents the findings of the regression analysis, where each row delineates the regression coefficient and its associated *t* value for an individual independent variable. Each column corresponds to a distinct regression model, denoted by the dependent variable, and specifies the matching pattern and sample size. To further validate the effect of CEM matching, column (1) of Table 4 shows the linear regression results with fundraiser race as the dependent variable, whereas column (2) shows the degree of regression effect of each covariate on race in the CEM-matched sample. All covariates in the nonmatched sample were significant. However, after matching, as expected, none of the variables remained significantly correlated with the fundraiser race (White or people of racial and ethnic minorities), as seen in the CEM-matched sample where the regression excludes the effect of high correlation and multicollinearity between the independent and control variables. On the basis of this, we conducted linear and logistic regressions using average donation amount, donation counts, and funding success as dependent variables.

Table 4 shows the results of the regression models with fundraiser race and covariates estimating the 3 outcome measures in the matched sample. The dependent variables for average donation amount and donation number were analyzed using linear regression, whereas the dependent variable for funding success was analyzed using logistic regression. Owing to the large N, we set the cutoff for significance at *P*<.001. For average donation amount, all variables except gender (*P*=.04) and the length of campaign description (*P*=.62) were significant predictors. The regression coefficient for the race of the fundraiser was 7.26, indicating that it had a large effect on the average amount of donations, suggesting that White people received significantly higher average donations than fundraisers of other races. Although the race of the fundraiser did not show a significant association with the number of donations (*P*=.42), several covariates were found to be significant (*P*<.001) predictors of number of donations, with higher initiator age associated with fewer donations, and greater length of campaign description and higher goal amounts related to a higher number of individual donations.

Table 4 also shows racial differences in funding success, with a significant association between the race of crowdfunding campaign sponsors and meeting or exceeding campaign funding goals (*P*<.001). However, the success rate of medical crowdfunding campaigns generally is not high, with only 12,338 successful campaigns in this study’s sample, accounting for only 11.77%. Approximately 90% of the campaigns failed to achieve their fundraising goals. Among the successful crowdfunding campaigns, 7808 (63.28%) campaigns were initiated by White people, which was higher than the proportion of White people in the overall sample. Thus, after controlling for the influence of the covariates (all significant except gender), campaigns initiated by White people were likely to generate higher average donations than individuals of other races, and they were overrepresented among successful cancer crowdfunding campaigns.

To further explore the marginal values for different races on the average amount per donation, the independent variable fundraiser race was set as a multivariate variable, including Asian, Black, American Indian or Alaska Native, and White, with Asian as the reference group.

Table 5 presents the predicted marginal values of the average donation amount raised by fundraisers of various races while holding other variables constant at their mean values. The results indicate that when factors such as gender, age, and length of campaign description were held constant at the mean, the predicted marginal value of Asian fundraisers was US $79.09. The average donation amount predicted for Black fundraisers was the lowest, at US $73.19. Native American fundraisers had a predicted marginal value of US $74.43, and White fundraisers had the highest predicted marginal value at US $86.14. On the basis of the summary statistics provided in Table 2, the average donation amount across all groups was US $82.87. Only the average donation amount for White crowdfunding campaigners exceeds this value. The difference between the predicted marginal values for Black and White people in terms of the average fundraising amount was US $12.94, accounting for approximately 17.68% of the average value. This difference demonstrates a significant racial disparity in fundraising outcomes.

**Figure 1:**
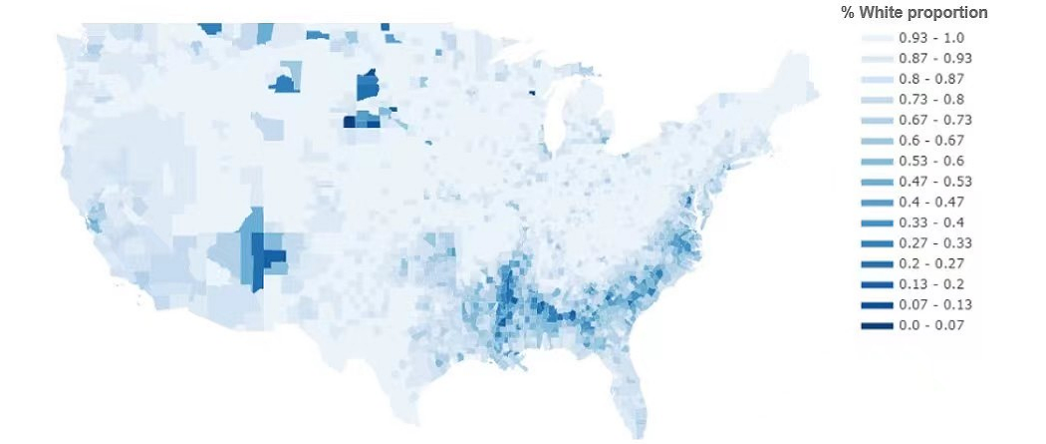
Proportion of White people in American counties.

Table 6 shows the parameter estimation results for predicting the average donation amount, including the multiplicative interaction effects. As can be seen from the results, there is no moderating effect of the gender of the fundraiser (*P*=.99) and age of the fundraiser (*P*=.82) on the relationship between race and average donation amount; however, the interaction of initiator race with the proportion of White people in the county is significant for the regression coefficient of the average donation amount (*P*<.001). Figure 1, based on data from the National Bureau of Economic Research for 2015, shows the percentage of White people in each county in the United States. The darker the color, the lower the percentage of White people in that county. The racial distribution is uneven, and overall, there are more counties with a high percentage of White people (Tables 7 and 8).

**Figure 2:**
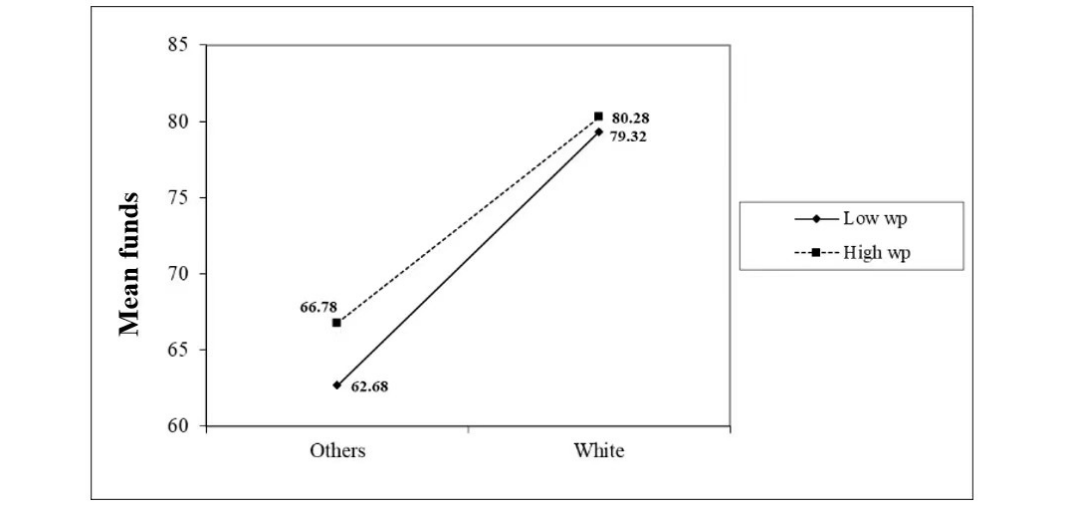
The moderating effect of a county’s White population on the impact of White versus non-White fundraisers on mean donation amount.

In Table 4, we observed that the higher the proportion of White people in the county, the higher the average donation amount with a regression coefficient of 6.600. In Table 6, the significant multiplicative interaction effect of race of fundraiser×proportion of White people in the county (*P*<.001) indicates that the proportion of White people in the fundraiser’s location has a moderating effect on the relationship between the race of the fundraiser and the average donation amount. The coefficient value of this variable (*P*<.001) indicates that the proportion of White people in the location of the fundraiser diminishes the effect of the race of the fundraiser on the mean value. Figure 2 shows the moderating effect, illustrating that when the proportion of White individuals in a location is low, the impact of fundraiser race on average fundraising is more pronounced. Conversely, when the White population is larger, the difference in mean fundraising values between White individuals and other racial groups narrows and the influence of race is weaker. However, in both cases, the average value for White fundraisers remains notably higher than for fundraisers by individuals from other racial groups, confirming racial disparities in average donation amount. In addition, regardless of the race of the sponsor, a higher proportion of White individuals in the county of residence of the campaign initiator corresponded to increased average donations.

**Table 4:** Results of regression analysis: the impact of race on fundraising outcomes.

Variable		Race (N=104,809)	Race CEM^a^ (n=77,553)	Average donation amount CEM (n=77,553)	Donation number CEM (n=77,553)	Funding success CEM (n=77,553)
**Race (1=White, 0=people of racial and ethnic minorities)**						
	Coefficient (SE)	N/A^b^	N/A	7.258 (15.05)	0.319 (0.42)	0.014 (6.33)
	*P* value	N/A	N/A	<.001	.42	<.001
**Gender (1=m** **an** **, 0=** **woman** **)**						
	Coefficient (SE)	−0.103 (−31.33)	0.000 (0.02)	0.904 (1.70)	−0.263 (−0.31)	0.003 (1.17)
	*P* value	<.001	.56	.04	.18	.12
**Age (y)**						
	Coefficient (SE)	0.005 (22.02)	0.000 (0.78)	0.585 (14.41)	−0.893 (−13.86)	−0.001 (−7.78)
	*P* value	<.001	.24	<.001	<.001	<.001
**Local White proportion**						
	Coefficient (SE)	0.249 (26.49)	−0.001 (−0.10)	6.600 (3.59)	−4.014 (−1.38)	−0.020 (−2.31)
	*P* value	<.001	.31	<.001	.57	.07
**Length of campaign description**						
	Coefficient (SE)	0.000 (1.04)	0.000 (1.14)	0.000 (0.59)	0.006 (13.50)	0.000 (15.42)
	*P* value	.41	.45	.62	<.001	<.001
**Goal amount**						
	Coefficient (SE)	0.000 (0.56)	−0.002 (−1.29)	12.854 (54.94)	25.322 (68.18)	−0.053 (−49.59)
	*P* value	.67	.71	<.001	<.001	<.001
**Creation date**						
	Coefficient (SE)	−0.000 (−10.45)	0.000 (0.21)	0.005 (5.97)	0.008 (5.58)	0.000 (9.79)
	*P* value	<.001	.23	<.001	<.001	<.001
**Constant**						
	Coefficient (SE)	1.622 (13.23)	0.554 (3.88)	−174.977 (−9.13)	−324.326 (−10.66)	−0.256 (−2.90)
	*P* value	<.001	<.001	<.001	<.001	<.001

^a^CEM: coarsened exact matching.

^b^N/A: not applicable.

**Table 5:** Marginal effect results of fundraisers’ race on the average donation amount.

Race	Values, mean (SD; SE)	*t* test (*df*)	*P*>|t|
Asian	79.08835 (72.80657; 0.5947056)	132.99 (15,409)	0.000
Black	73.19232 (82.77954; 0.8207497)	89.18 (8693)	0.000
American Indian or Alaska Native	78.05125 (62.05358; 0.639437)	122.06 (16,289)	0.000
White	86.13548 (72.22424; 0.2868712)	300.26 (64,390)	0.000

**Table 6:** The moderating effect of the county’s White population, gender, and age on the relationship between fundraisers’ race and average donation amount (N=104,809).

Variable	Coefficient	*t* test (*df*)	*P* value
Race	15.343	6.77 (104,806)	<.001
Local White proportion	7.854	3.61 (104,806)	<.001
Race × local White proportion	−9.921	−3.49 (104,806)	<.001
Constant	72.265	42.17 (104,806)	<.001

**Table 7:** The moderating effect of gender on the relationship between fundraisers’ race and average donation amount (N=104,809).

Variable	Coefficient	*t* test (*df*)	*P* value
Race	7.790	8.75 (104,806)	<.001
Gender	1.089	1.29 (104,806)	.21
Race × gender	−0.024	−0.02 (104,806)	.13
Constant	77.472	104.83 (104,806)	<.001

**Table 8:** The moderating effect of age on the relationship between fundraisers’ race and average donation amount (N=104,809).

Variable	Coefficient	*t* test (*df*)	*P* value
Race	6.695	2.95 (104,806)	<.001
Age (years)	0.545	10.34 (104,806)	<.001
Race × age	0.015	0.23 (104,806)	.48
Constant	60.521	34.44 (104,806)	<.001

### T Test Results

Focusing on White fundraisers, having their racial information identifiable in campaign images was associated with significantly higher average donation amounts and donation counts than for campaigns without racial cues (from any racial group), but not with higher funding success rates. Thus, the data in Table 9 suggest that White fundraisers are advantaged by disclosing racial information compared with individuals who do not disclose racial cues.

In contrast, for people of color, the disclosure of racial cues is associated with poorer funding outcomes compared with those who do not disclose racial cues in their campaigns. As presented in Table 10, the results indicate that for people of color, the disclosure of their racial identity information to potential donors is associated with lower average fundraising amounts and success rates. Other studies also found that exposure to racial cues and racial hints on web-based platforms may lead to worse fundraising outcomes, whereas racial anonymity in commercial crowdfunding platforms leads to higher success rates, particularly in reward campaigns [24].

**Table 9:** Independent sample *t* test results comparing White and nonracial matched samples.

	*t* test (*df*)	*P* value (2-tailed)	Mean difference^a^
Average donation amount	10.474 (104,802)	<.001^a^	4.574
Donation counts	3.329 (104,802)	.001^a^	5.474
Funding success	0.189 (104,802)	.85	.01

^a^Nonracial sample means images do not identify the fundraiser’s race or ethnicity.

**Table 10:** Independent sample *t* test results comparing people of racial and ethnic minorities and nonracial matched samples.

	*t* test (*df*)	*P* value	Mean difference^a^
Average donation amount	−6.497 (104,802)	<.001	−3.07386
Donation counts	5.137 (104,802)	<.001	6.9862
Funding success	−8.525 (104,802)	<.001	−0.018

^a^Nonracial sample means images do not identify the fundraiser’s race or ethnicity.

## Discussion

### Principal Findings

The purpose of this study was to examine racial differences in medical, specifically cancer, crowdfunding success and discuss the potential effect of bias on differences in the average donation amount, donation count, and funding success rate (goal amount reached or exceeded). Our results indicate that despite crowdfunding’s intent to support disadvantaged groups, significant disparities exist among different racial groups’ ability to benefit from cancer crowdfunding for 2 of the 3 funding outcome variables. In the following paragraphs, we discuss the effects of providing visual racial identifiers on these outcomes, the effect of the campaign initiator’s race, differences in the average amount of donations between different racial groups, and the moderating effect of the percentage of White people in the fundraiser’s county of residence, as well as their gender and age.

On web-based crowdfunding platforms, fundraisers can control whether they disclose their racial information. Identity cues can be directly verbalized, such as “I am an African-American and want to seek help,” or indirectly inferred through images, especially those with clear racial expressions, where potential donors can identify the race of the fundraiser by skin color and facial attributes [55]. Moreover, 19% of our sample disclosed racial cues in their images. We focused on these because they are the first impression of the campaign initiator to which potential donors will respond.

The results suggest that providing racial information may have differential effects for different racial groups, which we tested in our additional analyses. For White people, the recognition of racial information in campaign images leads to higher individual donation amounts, higher donation counts, and higher project success rates in medical crowdfunding. In contrast, for the individuals from other racial groups, the identification of their racial identity information by potential donors results in lower average funding and success rates. However, the presence of racial cues had a positive effect on donation counts. This is consistent with the findings from the study by Snyder et al [12] and suggests that more initial visual information about the campaign initiator helps build trust with potential donors, enhancing credibility that comes with self-representation of information, and thus, attracting more potential support. Nevertheless, a higher number of backers does not raise the average donation amount for people of color who disclose their race other above those with no racial information. On the basis of this, racial anonymity can be suggested as a means to improve racial discrepancies in funding.

Among individuals for whom race can be detected in their images, the race of people of color notably predicts funding outcomes, even after controlling for gender, age, percentage of White individuals in the fundraiser’s county of residence, length of campaign descriptions, goal amount, and date created. In particular, these fundraisers receive lower average donation amounts and have lower success rates than White fundraisers, although the number of donations does not differ between the groups. In addition, Black fundraisers receive the lowest amount of funding compared with Asian, American Indian, and White campaign initiators. This is consistent with research on general medical crowdfunding and reward campaigns, showing that the algorithms and technologies of web-based social networking platforms are disproportionately detrimental to historically marginalized groups [27,29,30,56]. We replicated this finding in cancer-specific crowdfunding. For marginalized racial groups, exposure to racial cues and racial hints on web-based platforms may lead to poorer funding outcomes, whereas racial anonymity in commercial crowdfunding platforms leads to higher success rates [22]. Given that the race of the fundraiser significantly impacts average donation amounts and whether the fundraising goal is met, without significant influence on the donation counts, there is reason to believe that donors are not simply rejecting fundraisers of color, but that a discounting is occurring, as roughly equal numbers of donors exhibit support for different racial groups, albeit with varying degrees of assistance. On the basis of the analysis of campaign texts containing racist and sexist tropes of selective deservingness by Davis et al [30], we do not suggest that implicit or explicit racism and bias no longer contribute to disparities in crowdfunding. However, although donors are likely affected by unconscious racial bias, leading them to donate smaller sums, it is also possible that individual donors to campaigns by people of color are less able to donate large amounts.

This notion is supported by our finding that a higher White population in the fundraiser’s area can mitigate racial discrepancies in average funding amounts. Most crowdfunding projects currently collect donations from existing social networks [57]. For fundraisers of various racial and ethnic backgrounds, the size of their potential donor networks is fairly comparable. Disparities in return may be related to systematic differences in discretionary income between White and donors and donors of other races [31,32]. According to data from the Federal Reserve, the average Black and Hispanic or Latino households in the United States earn approximately half as much as the average White household and own only approximately 15%-20% as much net wealth [58]. Financial inequality in neighborhoods has been proposed as a driver of inequality in cancer crowdfunding campaigns, with campaigns located in US counties with high socioeconomic status receiving greater total funds than campaigns in counties with lower socioeconomic status [59]. The most notable racial discrepancy in our data lies in the average donation amount. If the fundraisers who are not White primarily attract donors who are of the same race and who may not have substantial financial resources, then the characteristics of donor networks may matter as much or more than direct racial bias to the fundraising outcomes. Indeed, Igra et al [60] found that in wealthier counties, where people have greater educational achievement, campaigns for health and economic funding during COVID-19 received more funding than campaigns in areas with lower income and education. Owing to systemic racism, these areas are often White in the majority. In our data, the higher percentage of White individuals in the fundraiser’s county of residence is associated with a higher average donation amount, which reflects the financial ability of potential donors. The lower amounts that Black, Asian, and Native American campaigns receive per donation are not only related to their race but also to the racial distribution of their location, with people of color in areas with a higher percentage of White people receiving higher average donations. It is possible that, for these initiators, living in a majority-White area increases the likelihood of receiving donations from friends and neighbors with higher economic levels. Thus, geography can dilute racial discrimination to a certain extent. Whether this was the case in our sample can only be determined, however, by obtaining racial and socioeconomic identifiers from donors, a worthwhile goal for future research.

One alternative explanation from social psychology may also be relevant here. In counties with a smaller White population share, White individuals may experience these demographic shifts toward greater diversity as threatening to their status or group position [61]. White individuals living in areas with smaller percentages of other White individuals are more likely to report a perceived threat, have more negative biases toward racial groups different from their own, and have less support for racial integration [62-64]. Such attitudes may also be related to lower levels of generosity about support for government help [65] and in the context of donations [66].

It is also noteworthy that the campaigns initiated by people of color have more extreme rightward values for donation counts compared with the other 2 outcomes, with the highest donor count reaching 32,320 (data not shown). Thus, although White fundraisers’ crowdfunding campaigns receive significantly higher average donation amounts, thus incurring higher success rates, initiatives by Black, Asian, and American Indian cancer patients may garner a greater variety and sometimes a larger number of small donors.

In summary, our findings are consistent with previous work suggesting racial discrimination in medical crowdfunding and also with findings that suggest the importance of fundraisers’ network characteristics, such as education, average income, and donors’ race.

### Limitations

Our use of DeepFace to identify race represents both a limitation and a strength. As the racial information contained within the cover images may not be accurate or comprehensive, and the software cannot always discern whether cover images represent the campaign beneficiary, this may impact our interpretation of the research results. As the first impression perceived by potential donors, we nevertheless see the cover image as a stronger indicator of the impact of racial bias than self-identifying information in the text. The use of software rather than by-hand coding allows for the analysis of larger data sets.

Another limitation is the use of materials from only one platform, GoFundMe, which may limit the generalizability of our results to individuals who do not use or visit this platform, or any web-based platform, for assistance. Limitations owing to the constraints of the fundraising platform, where fundraisers’ personal information is identified by code also exist. Some campaign information anticipated for this study could not be obtained because of incomplete data. In the future, incorporating a manual review process could enhance the campaign’s rigor and further improve research.

Finally, a more definitive answer to the question of interpersonal racial bias versus racial disparities owing to disadvantaged networks will require additional data on the characteristics of actual donors. The argument that network characteristics are the true driver of lower campaign success requires characterizing the donors themselves, rather than proxy Facebook networks [32] or neighborhood deprivation [59], because many fundraisers’ networks extend beyond their immediate geographical surroundings.

### Conclusions

As a way for disadvantaged people to seek financial assistance in cancer care, crowdfunding appears to provide an easy-to-use platform for thousands of fundraisers. However, racial discrimination and network inequality both interfere with the utility of crowdfunding to affect social equality. On the basis of our findings, measures to reduce prejudice in crowdfunding may include maintaining racial anonymity and offering strong evidence for *deservingness,* particularly for the most disadvantaged groups. Racialized perceptions of deservingness continue to provide barriers to equality, but ongoing changes in public opinion, such as those seen after the 2020 Black Lives Matter protests [67], may begin to mitigate crowdfunding disparities.

If the unequal distribution of resources on social networks is to blame, then efforts to reach more racially diverse networks with a high percentage of White or wealthier individuals can further reduce fundraising disparities. The most ideal, of course, would be institutional and societal change that eliminates the financial distress in health care, which causes people to turn to their networks for financial support in the first place.

## Abbreviations

CEM: coarsened exact matching
